# Survival time following resection of intracranial metastases from NSCLC-development and validation of a novel nomogram

**DOI:** 10.1186/s12885-017-3763-x

**Published:** 2017-11-21

**Authors:** Xiaoyu Ji, Yingjie Zhuang, Xiangye Yin, Qiong Zhan, Xinli Zhou, Xiaohua Liang

**Affiliations:** 1 Department of oncology, Huashan Hospital Fudan University, Shanghai, 200040 China; 2 Company 4, Battalion 1, Cadet Brigade 1, Fourth Military Medical University, Xi’an, 710032 China

**Keywords:** Non-small-cell lung cancer, Brain metastases, Prognostic indexes, Intracranial surgery, Nomogram

## Abstract

**Background:**

Brain metastases (BM) from non-small cell lung cancer (NSCLC) are the most frequent intracranial tumors. To identify patients who might benefit from intracranial surgery, we compared the six existing prognostic indexes(PIs) and built a nomogram to predict the survival for NSCLC with BM before they intended to receive total intracranial resection in China.

**Methods:**

First, clinical data of NSCLC presenting with BM were retrospectively reviewed. All of the patients had received total intracranial resection and were randomly distributed to developing cohort and validation cohort by 2:1. Second, we stratified the cohort using a recursive partitioning analysis(RPA), a score index for radiosurgery (SIR), a basic score for BM (BS-BM), a Golden Grading System (GGS), a disease-specific graded prognostic assessment (DS-GPA) and by NSCLC-RADES. The predictive power of the six PIs was assessed using the Kaplan–Meier method and the log-rank test. Third, univariate and multivariate analysis were explored, and the nomogram predicting survival of BMs from NSCLC was constructed using R 3.2.3 software. The concordance index (C-index) was calculated to evaluate the discriminatory power of the nomogram in the developing cohort and validation cohort.

**Results:**

BS-BM could better predict survival of patients before intracranial surgery compared with other PIs. In the final multivariate analysis, KPS at diagnosis of BM, metachronous or synchronous BM and the histology of lung cancer appeared to be the independent prognostic predictors for survival. The C-index in the developing cohort and validation cohort were 0.75 and 0.71 respectively, which was better than the C-index of the other six PIs.

**Conclusions:**

The new nomogram is a promising tool in further choosing the candidates for intracranial surgery among NSCLC with BM and in helping physicians tailor suitable treatment options before operation in clinical practice.

## Background

Brain metastases (BM) are the most frequent intracranial tumors, resulting in significant morbidity and mortality. Among these patients, non-small cell lung cancer (NSCLC) ranks as a leading cause. As a result of prolonged overall survival(OS) in NSCLC patients and better detection of subclinical lesions, incidences of BM are increasing [1]. The risk of developing BM in advanced NSCLC (stage III-IV) is approximately 30%–50%. Even in resected early stage patients (stage I-II), the risk of developing BM at 5 years is 10% [2].

Until recently the median survival time (MST) for patients with BM was still not good [3]. BM is a highly heterogeneous disease, and prognosis and treatment options should be determined depending on the patient’s performance status, the number, size and location of BM, the pathologic type, and the control of the primary tumor and extracranial disease. Some candidates decided to receive surgery if intracranial lesions could be totally resected. In clinical practice, only a portion of those candidates could benefit from the intensive treatment. There have been few studies on how to further identify those candidates who might benefit from surgery, and the individuals should avoid overtreatment before they decided to receive intracranial surgery.

Many prognostic indexes (PIs) for predicting the prognosis of BM have been developed based on retrospective studies [4]. In 1997, the Radiation Therapy Oncology Group established the first prognostic score called the recursive partitioning analysis (RPA) [5]. Then, the Score Index for Radiosurgery (SIR) [6], the basic score for BM (BSBM) [7], the Golden Grading System (GGS) [8], the disease-specific graded prognostic assessment (DS-GPA) [9] and the NSCLC-RADES [10] emerged (the details of the six PIs are shown in Table 1). The published PIs have been used to help physicians tailor suitable treatment options based on the prognosis prediction. However, they were mostly designed for BM patients who were treated with radiotherapy. Whether patients who received intracranial surgery as first line treatment can be stratified by the PIs is not known.

A nomogram is a graphical prediction model widely used to predict cancer prognosis. It combines several prognostic factors on the basis of the Cox proportional hazards model and reduces statistical predictive models into a single numerical estimate of the probability of an event, such as death or recurrence [11]. As a result, an individual prediction of a specific outcome can be provided for each patient. In this study, we analyzed a cohort of patients retrospectively, compared the prediction ability of six PIs, and developed a new nomogram to identify the NSCLC patients presenting with BM who might benefit from intracranial surgery more precisely and help physicians tailor more suitable treatment options.

**Table 1:** Six prognostic indexes for patients with non-small cell lung cancer with brain metastases

Prognostic factors	RPA	SIR	BS-BM	GGS	DS-GPA	NSCLC-RADES
Sample	1200	65	110	479	5067	514
Age(years)	<65/≥65	≤50(2′), 51–59(1′), ≥60(0′)	_	≥65(1′), <65(0′)	<50(1′), 50–60(0.5′), >60(0′)	
gender						M (2′), F (5′)
KPS (%)	≥70/<70	80–100(2′), 60–70(1′), ≤50(0′)	80–100(1′), ≤70(0′)	<70(1′), ≥70(0′)	90–100(1′), 70–80(0.5′), <70(0′)	<70(1′), ≥70(5′)
CPT	Y/N		Y (1′), N (0′)			
ECM	Y/N	CR (2′), PR/stable (1′), PD (0′)	N (1′), Y (0′)	Y (1′), N (0′)	N (1′), Y (0′)	Y (2′), N (5′)
Vol. of BM(cm^3^)	_	<5(2′), 5–13(1′), >13(0′)	_	_	_	_
Number of BM	_	1(2′), 2(1′), ≥3(0′)	_	_	1(1′), 2–3(0.5′), >3(0′)	_
Class I	All 4 favorable factors	8–10’	3’	0’	0–1’	5–9’
Class II	others	4–7’	2’	1’	1.5–2’	11–12’
Class III	KPS < 70	1–3’	1’	2’	2.5–3’	15’
Class IV			0’	3’	3.5–4’	

*RPA* recursive partitioning analysis, *SIR* Score Index for Radiosurgery, *BS-BM* basic score for BM, *GGS* Golden Grading System, *DS-GPA* disease-specific graded prognostic assessment, *CPT* control of primary tumor, *ECM* extracranial metastases, *BM* brain metastases, *Y* yes, *N* no, *M* male, *F* female, *KPS* Karnofsky performance status, *CR* complete response, *PR* partial response, *PD* progressive disease

## Methods

### Patients

We collected the data of 335 NSCLC patients presenting with BM between 01/2003 and 12/2009. All of the patients were diagnosed and treated at Huashan Hospital, Fudan University, Shanghai, China. They were randomly distributed to developing cohort and validation cohort by 2:1. The inclusion criteria was histologically confirmed BM from NSCLC, and BM lesions not exceeding three to ensure that they received total intracranial resection. Exclusion criteria were patients with leptomeningeal metastases (meningeal enhancement on MRI or tumor cells found in cerebral spinal fluid), and either histological or clinical evidence of other malignant tumors except NSCLC.

### Data collection and follow-up

The data from the medical records included: age, gender, the KPS at the time of BM diagnosis, the time of the primary and metastatic tumor diagnosis, the pathology type of the tumor, the presence of extracranial metastases, the control of primary tumor, and brain involvement characteristics. Synchronous BM was defined as lesions in the brain that were detected within three months of NSCLC diagnosis. Metachronous BM was defined as there have been no evidence of BM within three months of the NSCLC diagnosis.

The follow-up was by phone-call or letter. All patients were followed until death or up to May 1, 2015. The information included: 1) follow-up treatments; 2) survival data; and 3) the date of death.

### Statistical analysis

The primary end-point was OS, defined as the interval from the date of BM diagnosis to the date of death or failure of follow-up. Patients alive without events were censored at the end of the follow-up. The diagnosis of BM needed to be confirmed by at least two experienced pathologists. Two hundred and twenty-three patients were distributed to the developing cohort randomly and the other one hundred and twelve patients were distributed to the validation cohort. The developing cohort was stratified by RPA, SIR, BS-BM, GGS, DS-GPA, and NSCLC-RADES. The OS curves were drawn by subgroups of the six PIs. OS was estimated by the Kaplan–Meier method, and the MST of each subgroup was compared among subgroups using the log-rank test. Harrell’s concordance Index (C-index) was used to assess the discriminating ability of the six PIs. The value of C-index ranges between 0.5 and 1. 0.5 represents completely inconsistent with the practical situation, indicating that the nomogram has no predictive effect; 1 means the predictive result of the nomogram is exactly the same with the practical situation. Prognostic factors found to be *p* < 0.1 on univariate analysis were further explored in a multivariate analysis used with the Cox proportional hazards model. The significant variables (*p* < 0.05 in the multivariable Cox model) were seen as prognostic factors in the final nomogram. The new nomogram predicting the prognosis of NSCLC presenting with BM was also measured by C-index in the developing cohort and validation cohort. we used the bootstrap-corrected C-index to measure discriminative ability of the nomogram.

The statistical analyses were calculated with SPSS Statistics23.0 (IBM, SPSS Inc. Chicago, IL, US) and R 3.2.3 software (https://www.r-project.org/).

## Results

### The developing cohort patients’ characteristics

In the developing cohort, a total of 223 patients were qualified for the retrospective study. By May 1, 2015, all enrolled patients arrived at the end point, apart from the 25 individuals lost during the follow-ups and the 7 patients still alive. One hundred and sixty patients received only a gross total resection, and the others were treated in combination with whole brain radiation therapy (WBRT) or stereotactic radiation (SRS). The differences of MST between the only operative group and the postoperative radiation therapy group showed no statistical significance (*p* = 0.260). Most patients were male and the median age was 58 years (range 22–85 years). In the metachronous entity, the intervals from NSCLC diagnosis to the confirmation of BM ranged from 3 to 68 months. Detailed characteristics of patients are listed in Table 2.

**Table 2:** Characteristics of the developing cohort patients and the validation cohort patients with brain metastases from non-small cell lung cancer

Characteristics	Developing cohort (*n* = 223)		Validation cohort (*n* = 112)
	*N* (%)	*p* value	*N* (%)
Gender		0.013	
Male	144 (64.6%)		73 (65.2%)
Female	79 (35.4%)		39 (34.8%)
Age of BM diagnosis(years)		0.311	
< 60	134 (60.1%)		61 (54.5%)
≥ 60	89 (39.9%)		51 (45.5%)
KPS (%)		<0.001	
≥ 80	99 (44.4%)		45 (40.6%)
< 80	124 (55.6%)		67 (59.4%)
Interval from NSCLC diagnosis to BM diagnosis		0.044	
Synchronous	159 (71.3%)		86 (76.8%)
Metachronous	64 (28.7%)		26 (23.2%)
Time from neural symptom onset to BM diagnosis(months)		0.759	
≤ 1	159 (71.3%)		81 (72.3%)
1–3	45 (20.2%)		25 (22.3%)
> 3	19 (8.5%)		6 (5.4%)
ECM when BM diagnosis		0.009	
Yes	85 (38.1%)		35 (31.3%)
No	138 (61.9%)		77 (68.8%)
Number of BM		0.925	
1	148 (66.3%)		70 (62.5%)
2	11 (5.0%)		14 (12.5%)
3	64 (28.7%)		28 (25.0%)
Tumor size(cm)		0.348	
≤ 2	62 (27.8%)		26 (23.2%)
> 2	124 (55.6%)		67 (59.8%)
Unknown	37 (16.6%)		19 (17.0%)
Histology		0.006	
Adenocarcinoma	116 (52.2%)		75 (67.0%)
Squamous cell lung cancer	25 (11.2)		20 (17.9%)
Poorly differentiated carcinoma or histology can’t be distinguished	82 (36.8%)		17 (15.1%)

*BM* brain metastases, *KPS* Karnofsky performance status, *ECM* extracranial metastases

### Survival analysis and PIs comparison

**Fig. 1:**
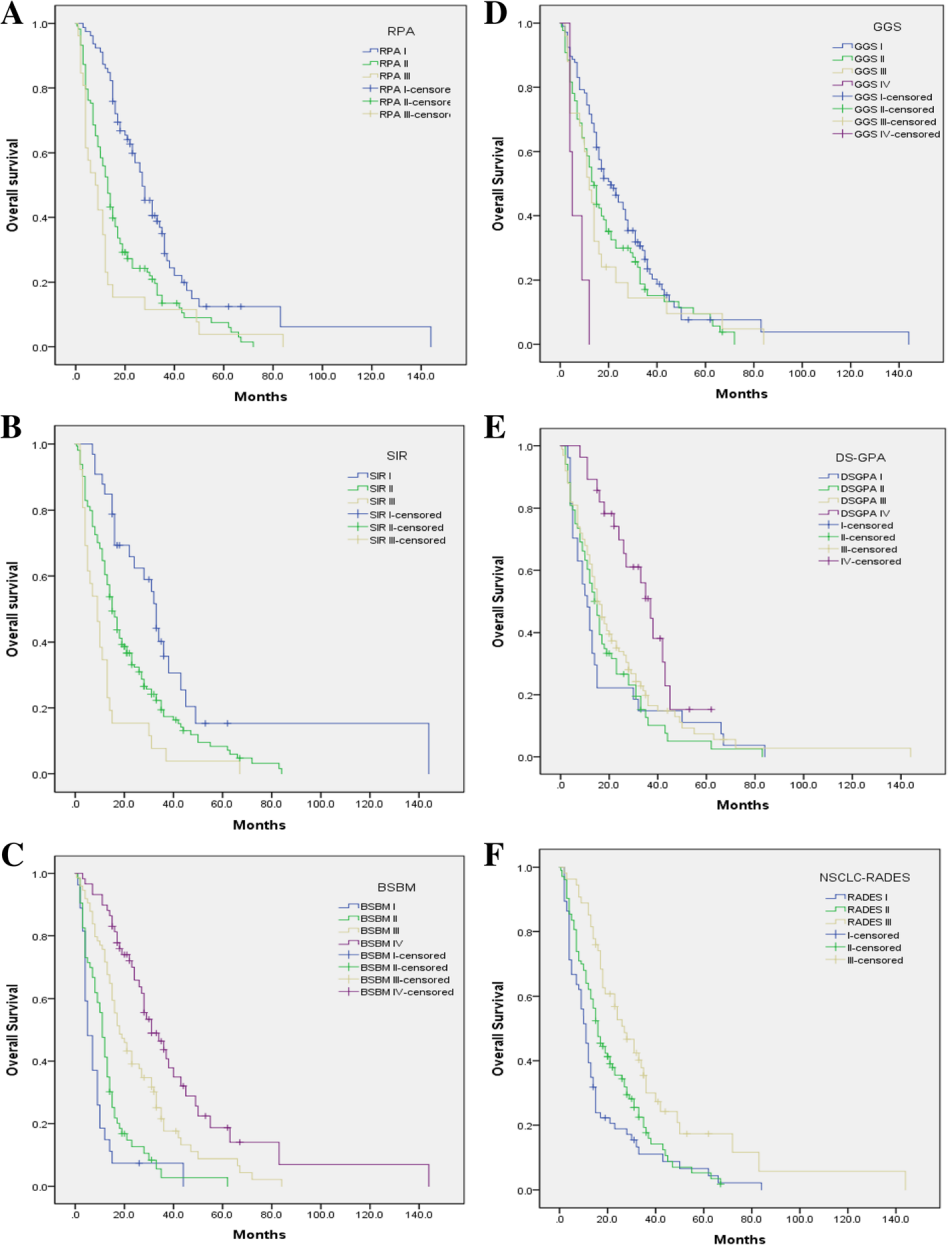
Overall survival curves of the developing cohort subgrouped by six different prognostic indexes. The picture **a**-**f** represents overall survival curves of the developing cohort subgrouped by RPA、SIR、BS-BM、GGS、DS-GPA and NSCLC-RADES. The predictive abilities of the six PIs are different

The MST of the developing cohort was 15 months (95% confidence interval, 13.01–16.99 months), and survival rates at 6-months, 1-, 2-, 3- and 5-years were 80.2%, 61.0%, 30.0%, 11.7% and 4.5% respectively. Population repartition and the MST in each subgroup are listed in Table 3. Survival curves were demonstrated in Fig. 1. All classes were represented by at least 10% of the patients, with the exception of class IV in the GGS. The results showed that the six PIs could discriminate with statistical significance (*p* < 0.05). However, differences of MST in some contiguous classes showed no statistical significance. MST of RPA class II and class III (*p* = 0.144), every adjacent classes of GGS (*p* = 0.058, 0.631, 0.054 respectively), DS-GPA class I and class II (*p* = 0.799), DS-GPA class II and class III (*p* = 0.261) could not be discriminated well. Only SIR, BS-BM and NSCLC-RADES had statistical significance between every adjacent subgroup.

**Table 3:** Distribution of the population and MST for each PI

PI	Number of patients	%	MST (months)	*p* value
RPA			15	<0.001
I	79	35.4	27	<0.001
II	118	52.9	13	0.144
III	26	11.7	8	
SIR			15	<0.001
I	33	14.8	33	0.003
II	164	73.5	15	0.003
III	26	11.7	9	
BS-BM			15	<0.001
I	27	12.1	5	0.036
II	63	28.3	11	<0.001
III	74	33.2	18	0.001
IV	59	26.4	31	
GGS			15	0.001
I	106	47.5	21	0.058
II	87	39.0	14	0.631
III	25	11.2	12	0.054
IV	5	2.3	5	
DS-GPA			15	0.003
I	27	12.1	11	0.799
II	68	30.5	14	0.261
III	100	44.8	15	0.003
IV	28	12.6	37	
NSCLC-RADES			15	<0.001
I	66	29.6	11	0.025
II	103	46.2	16	0.002
III	54	24.2	27

To further evaluate the discriminatory power of the six PIs, we calculated the C-index using R software. The C-index value of BSBM was 0.69, higher than the other five PIs (RPA: 0.64, SIR: 0.59, GGS: 0.58, DS-GPA: 0.59, NSCLC-RADES: 0.62).

### Univariate and multivariate analysis

In the univariate analysis of the possible prognostic factors, we considered the nine variables listed in Table 2, and the following five factors, female (*p* = 0.013), KPS ≥80 (*p* < 0.001), metachronous (*p* = 0.044), absence of ECM (*p* = 0.009), and histology of lung adenocarcinoma (*p* < 0.001) were associated with prolonged OS. The final multivariate analysis is shown in Table 4 *.* Independent prognostic predictors for better survival were KPS ≥80 at diagnosis of BM, metachronous BM and the histology of lung adenocarcinoma.

**Table 4:** Multivariate analysis of prognostic factors

Prognostic factors	HR	95% CI	*P* value
Gender (male/female)	1.297	0.954–1.762	0.097
KPS (<80/≥80)	2.087	1.539–2.831	<0.001
Metachronous/Synchronous	0.685	0.489–0.961	0.028
ECM (N/Y)	0.749	0.054–1.012	0.060
Histology (non-adenocarcinoma/adenocarcinoma)	1.303	1.114–1.524	0.001

*MST* median survival time, *KPS* Karnofsky performance status, *ECM* extracranial metastases

### Establishment and validation of the nomogram

**Fig. 2:**
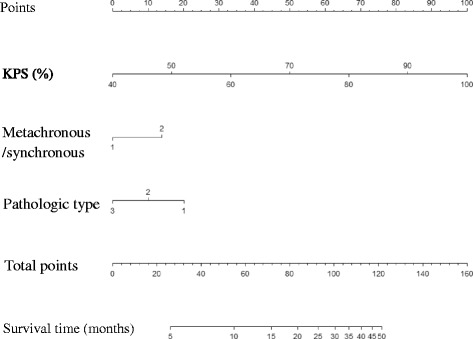
Nomogram for predicting survival time of NSCLC with brain metastases. To obtain the estimated survival time of each individual patient, we determined the value for each variable by drawing a vertical line to the points scale, then summed up the three values and drew a vertical line from the total points scale to the survival time scale. Note: Metachronous/synchronous (1- synchronous, 2- metachronous); Pathologic type (1- adenocarcinoma, 2- squamous carcinoma, 3- others)

Following the multivariable Cox model, the three independent variables, KPS at the diagnosis of BM, metachronous/synchronous BM, and the pathologic type of NSCLC were selected in the final nomogram to predict the survival time of NSCLC presenting with BM before they decided to receive complete surgical resection. The nomogram was shown in Fig. 2.

One hundred twelve patients were included in the validation cohort, whose characteristics were similar to the counterpart in the developing cohort. They were also followed until May 1, 2015. All enrolled patients arrived at the end point, apart from the 5 individuals lost during the follow-ups and the 2 patients still alive. The median OS of the validating cohort was 15 months (95% confidence interval, 9.70–16.30 months), and the survival rates at 6-months,1-, 2-, 3- and 5-years were 77.7%, 51.0%, 27.4%, 13.2% and 5.7% respectively. Most patients were male and the median age was 58 years (ranging 38–80 years). Table 2 shows the detailed characteristics of the validation patients. The C-index for the developing cohort and the validation cohort were 0.75 and 0.71 respectively.

## Discussion

Brain metastases are becoming an increasingly common challenge for the clinician. The role of complete surgical resection in brain metastatic patients is still controversial [12]. Traditionally, the treatment for BM generally relied on radiotherapy and chemotherapy. Even if intracranial lesions could be totally resected, the survival time would not be extended [13]. Meanwhile, the operations themselves might result in higher mortality rates. However, with the advances in surgical techniques, patients with BM might benefit from intracranial operations, as confirmed by some studies.

Since the 1980s, more studies have emphasized the importance of surgery in treatment for BM. They compared intracranial operations with other treatments, like WBRT or SRS [14]. Although the results were not always consistent, it could be concluded that some patients benefit from intracranial operation [15–17]. Moreover, surgery allows a relief of intracranial hypertension, seizures and focal neurological deficits, and is the most useful way to get a clear pathologic diagnosis. Surgery has become an important therapeutic option for patients presenting with BM [16, 18]. As the NCCN guidelines recommend, for one to three brain metastatic lesions, and stable systemic diseases, surgical resection may be considered. However, clinical data show some eligible patients cannot benefit from intracranial operation whatsoever. Operative indications for BM are still ardently disputed. As such, identifying patients who might benefit from intracranial surgery more precisely and helping physicians tailor more suitable treatment options are crucial.

Currently, there is no research to compare the existing PIs in BM patients who were treated with intracranial total resection [19]. We enrolled 335 eligible patients in this study. Completely surgical resection of intracranial lesions was used as the first line treatment option. We eliminated the possibilities that different treatments may affect the survival outcome, and explored the relationship between baseline situations and the prognosis.

RPA [5] is commonly used in the prognosis prediction. It was developed in patients who were treated with WBRT. Agboola [20], once applied in a cohort of surgical resected BM patients, showed the predictive value of RPA. However, the 1200 enrolled patients came from three different trials, and the criteria and the dose of WBRT were not same. SIR [6] resulted in BM-related variables: the numbers and sizes of BM. Some studies found that patients benefitted from surgical treatment for BM. BSBM [7] has been advocated as a convenient, easy to use PI, which was proposed on the basis of RPA and SIR. It was further evaluated in patients receiving WBRT with surgery and WBRT with or without SRS [21]. GGS [8] was constructed specifically for NSCLC patients. However, it failed to distinguish a good prognosis from a poor prognosis in our study. DS-GPA [9] was proposed in a large sample multi-center retrospective study. With the enrolled patients spanning from 1985 to 2007, it could not eliminate the influence of treatments, and different criteria, treatment measures, and selection bias were unavoidable. The newly proposed NSCLC-RADES [10] needs to be further validated in more studies.

With the six PIs targeting different populations, we could not demonstrate that one prognostic classification was superior to the rest [22]. In our research, SIR, BSBM, NSCLC-RADES, especially BSBM better predicted the survival of BM from NSCLC who were treated with intracranial surgery in China. However, some patients were still misclassified to “good prognosis” and “poor prognosis” in BSBM. So the existing PIs are still not the ideal prognostic tool to help identify those patients who might benefit from intensive treatment like surgery, and the individuals should avoid overtreatments. The PIs need to be further optimized.

In our univariate and multivariate analyses, independent prognostic predictors for better survival were KPS at diagnosis of BM, metachronous BM and the histology of lung adenocarcinoma.

KPS at the BM diagnosis, which was also evaluated in the six studied PIs, was a significant prognostic factor in the study. Neurological symptoms, like headaches, motor impairment, dysphasia, seizures, and even coma, are always induced by intracranial lesions. Some discomfort, like coughing, sputum, and chest congestion are related to systematic cancer. All of these symptoms influence the KPS score and affect the prognosis. As a result, use of the KPS has been criticized because of its subjective nature, variability in scoring between observers, and the tendency for the score to be influenced by acute but self- limited events [23]. When we evaluate the variable, we should notice that and try to make KPS reliable. .

The pathological types of NSCLC were found to be a significant factor for prognosis, which was not involved in the six PIs. Lung adenocarcinoma (ADC) and squamous cell carcinoma (SCC) accounted for 80% of NSCLC. Our research showed significantly better OS for ADC. This result is in accordance with many other published studies [24]. There may be some reasons behind this phenomenon. First, the natural biological behaviors are not the same. The next-generation sequencing of the SCC subgroup identified entirely different genes [25]. Second, due to higher incidences of mutant genes (EGFR, ALK, ROS1, etc.) in ADC [26], the use of new targeted agents will enhance the response rates and prolong OS. We did not investigate the other rare types of NSCLC.

In 2012, our institution conducted a study to compare synchronous BM with metachronous BM. We found that the clinical characteristics, diagnoses, and treatment methods for synchronous BM and metachronous BM were different [24]. In our cohort, 73.1% of the patients were synchronous BM. As analyzed above, the MST in metachronous BM was longer than in the synchronous BM. The possible reasons for this are as follows: 1) control of primary tumor; 2) presence of ECM; 3) sizes of BMs; and 4) even dissimilitude driver genes of the two subgroups. Further research is needed to better understand these findings.

A nomogram is widely used for cancer prognosis, primarily because of its ability to integrate different variables on the basis of multivariate analysis to more accurately predict the survival of individuals. Kaizu [27] et al. established a nomogram to evaluate the risk of bone-metastasis in postoperative prostate cancer patients. Bevilacqua [28] developed a nomogram to predict the sentinel lymph node metastasis in early breast cancer and the survival of patients with breast cancer. Graesslin [29] even set up a nomogram to predict the incidence of brain metastasis in breast cancer. However, a nomogram for predicting the survival time of NSCLC patients with brain metastasis before they decided to receive complete surgical resection has not been previously investigated.

Our new nomogram is a predictive tool, which creates a simple graphical representation of a statistical predictive model to predict the survival time of individual NSCLC patient with brain metastasis for intracranial surgery. Through quantifying the risk of death with a variety of factors, the nomogram can help clinicians tailor treatment modalities and avoid good prognostic patients from giving up effective treatment and prevent the poor prognostic patients from receiving overtreatment. The C-index of the nomogram showed its superior ability to predict prognosis. In conclusion, before clinicians and NSCLC patients consider to have an intracranial resection surgery, our nomogram could be used as an effective tool to predict the survival of the patients and optimize treatment modalities in clinical practice.

Despite some findings of the present study, there are still several limitations. First, with the advent of targeted therapy, mutation testing has been standard practice with a NSCLC diagnosis. However, the gene expression patterns of our enrolled patients were unknown. As a result, we could not account for the molecular subtype. Although the efficacy of surgery may not be influenced by this factor, the patient’s gene status should be as clear as possible in further studies. Second, as a single institution retrospective study, treatment protocols, patient selection, and follow-ups can bias the results. For all of the patients in our cohort who received intracranial surgery, the factors of KPS, age, ECM, and number of BMs were better than the average. Third, future multicenter studies are needed to confirm our developed nomogram.

## Conclusions

In conclusion, we found that BS-BM could better predict survival of the BM patients after comparing the six existing PIs. In the final multivariate analysis, KPS ≥80 at diagnosis of BM, metachronous BM and the histology of lung adenocarcinoma appeared to be the independent prognostic predictors for better survival. Additionally, the new nomogram we built in the study is a predictive tool in further choosing the candidates for intracranial surgery among eligible NSCLC with BM. As a result, it helps to optimize NSCLC with BM patients’ treatment modalities in clinical practice.

## Abbreviations

ADC: Lung adenocarcinoma
BM: Brain metastases
BS-BM: Basic score for BM
C-index: Concordance index
CPT: Control of primary tumor
DS-GPA: Disease-specific graded prognostic assessment
ECM: Extracranial metastases
EGFR: Epidermal growth factor receptor
GGS: Golden Grading System
KPS: Karnofsky performance status
MST: Media survival time
NSCLC: Non-small cell lung cancer
OS: Overall survival
PIs: prognostic indexes
RPA: Recursive partitioning analysis
SCC: Squamous cell carcinoma
SIR: Score index for radiosurgery
SRS: Stereotactic radiation
WBRT: Whole brain radiation therapy
